# Supplementary figures and images for: A novel multi-word paradigm for investigating semantic context effects in language production
Source: PLoS One. 2020 Apr 10;15(4):e0230439. doi: 10.1371/journal.pone.0230439 (PMC7147796; doi:10.1371/journal.pone.0230439)

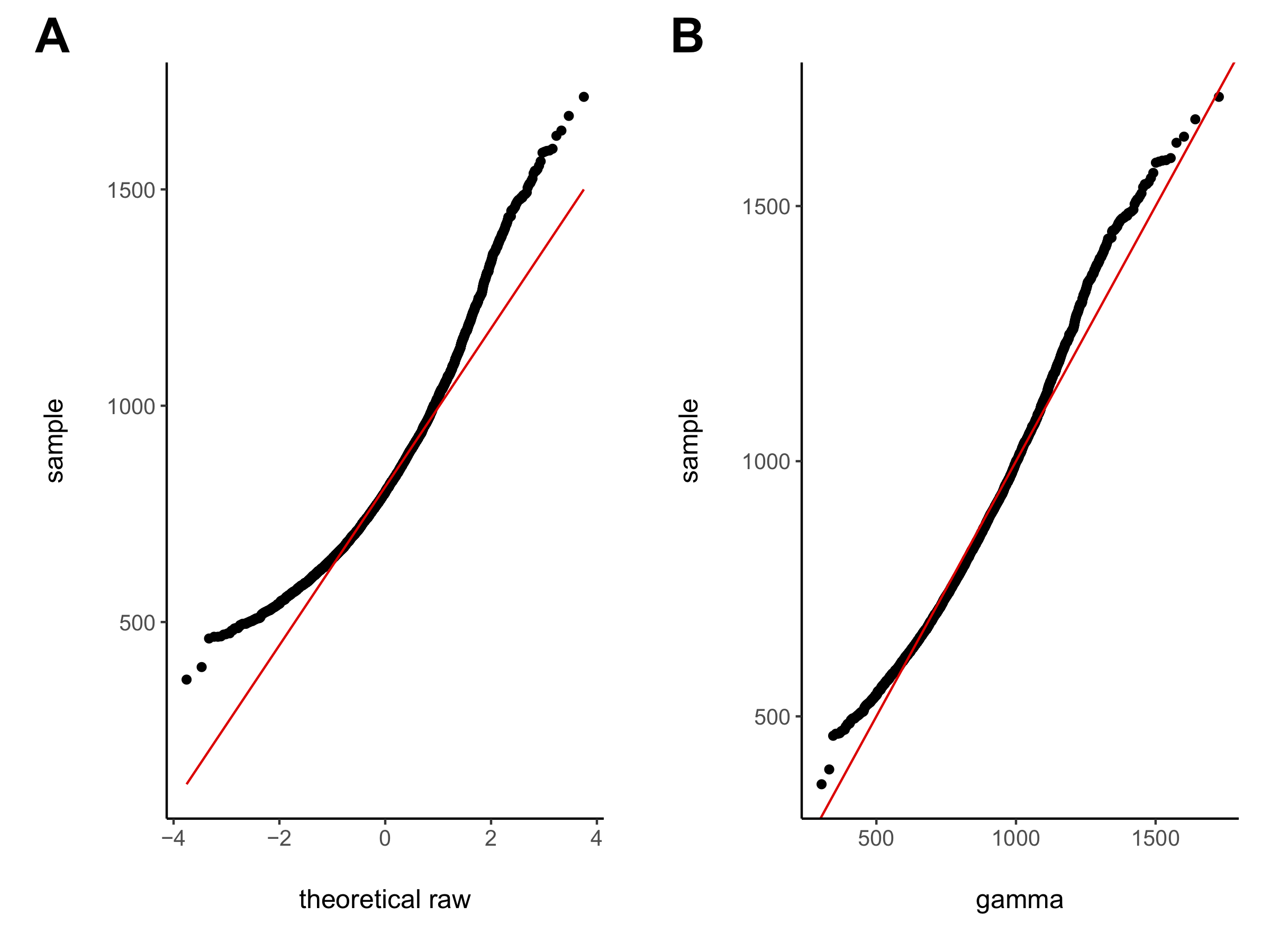

Supplement: S1 Fig — Panel A: raw RTs, panel B: RTs with gamma distribution. (TIFF) [file pone.0230439.s001.tiff]
