# Supplementary material for: A novel multi-word paradigm for investigating semantic context effects in language production
Source: PLoS One. 2020 Apr 10;15(4):e0230439. doi: 10.1371/journal.pone.0230439 (PMC7147796; doi:10.1371/journal.pone.0230439)
Supplement: S1 Appendix — (DOCX) [file pone.0230439.s003.docx]

Appendix A. Stimuli

| Category | Items |  |  |  |  |  |
| --- | --- | --- | --- | --- | --- | --- |
| hoofed animals | Reh  (deer) | Pferd (horse) | Esel  (donkey) | Schaf  (sheep) | Kamel (camel) | Ziege  (goat) |
| fruits | Apfel (apple) | Birne  (pear) | Traube (grape) | Erdbeere (strawberry) | Kirsche (cherry) | Orange (orange) |
| seating furniture | Sofa  (couch) | Stuhl  (chair) | Hocker  (stool) | Sessel (armchair) | Bank (bench) | Thron (throne) |
| carpenter's tools | Hammer (hammer) | Säge  (saw) | Schraube (screw) | Axt  (axe) | Zange (pliers) | Bohrer (drill) |
| face parts | Auge  (eye) | Nase  (nose) | Mund (mouth) | Ohr  (ear) | Kinn (chin) | Haare  (hair) |
| street vehicles | Auto  (car) | Lastwagen (truck) | Motorrad (motorcycle) | Kutsche (carriage) | Bus  (bus) | Traktor (tractor) |
| upper boddy clothing | Mantel (coat) | Jacke (jacket) | Pullover (sweater) | Hemd  (shirt) | T-Shirt  (t-shirt) | Bluse (blouse) |
