# Supplementary material for: A novel multi-word paradigm for investigating semantic context effects in language production
Source: PLoS One. 2020 Apr 10;15(4):e0230439. doi: 10.1371/journal.pone.0230439 (PMC7147796; doi:10.1371/journal.pone.0230439)
Supplement: S2 Appendix — (DOCX) [file pone.0230439.s004.docx]

Appendix B. Naming latencies

Table B1. GLMM for the effect of picture type and set size on RTs

| Term | Estimate | SE | t | p |
| --- | --- | --- | --- | --- |
| Intercept | 862.65 | 4.02 | 214.33 | <0.001 |
| Picture type: rel-unrel ^a^ | 10.94 | 4.00 | 2.73 | 0.006 |
| Set size: 4-3 | -11.33 | 4.13 | -2.74 | 0.006 |
| Set size: 5-3 | -11.79 | 4.05 | -2.91 | 0.004 |
| Pic type * set size: 4-3 | -11.98 | 5.21 | -2.30 | 0.021 |
| Pic type * set size: 5-3 | 0.46 | 5.87 | 0.08 | 0.938 |
| *Notes.* ^a^ henceforth “pic type”. | | | | |

Table B2. GLMM for the effect of picture type nested within the levels of set size

| Term | Estimate | SE | t | p |
| --- | --- | --- | --- | --- |
| Intercept | 862.73 | 4.77 | 180.70 | 0.000 |
| Set size 4-3 | -11.58 | 3.96 | -2.92 | 0.003 |
| Set size 5-3 | -12.05 | 3.72 | -3.24 | 0.001 |
| Set size 3: Picture type rel-unrel^a^ | 14.11 | 4.03 | 3.50 | 0.000 |
| Set size 4: Pic type rel-unrel | 2.78 | 3.94 | 0.70 | 0.481 |
| Set size 5: Pic type rel-unrel | 15.56 | 4.52 | 3.44 | 0.001 |
| *Notes.* ^a^ henceforth “pic type”. | | | | |
