# Supplementary material for: A novel multi-word paradigm for investigating semantic context effects in language production
Source: PLoS One. 2020 Apr 10;15(4):e0230439. doi: 10.1371/journal.pone.0230439 (PMC7147796; doi:10.1371/journal.pone.0230439)
Supplement: S3 Appendix — (DOCX) [file pone.0230439.s005.docx]

Appendix C. Fixation durations

Table C1. GLMM for the effect of word type and set size on fixation durations

| Term | Estimate | SE | t | p |
| --- | --- | --- | --- | --- |
| Intercept | 549.24 | 7.36 | 74.65 | <0.001 |
| Word type: rel-unrel* | 114.44 | 16.05 | 7.13 | <0.001 |
| Set size: 4-3 | -18.16 | 5.25 | -3.46 | 0.001 |
| Set size: 5-3 | -28.34 | 6.31 | -4.49 | <0.001 |
| Word type * set size: 4-3 | 13.84 | 12.60 | 1.10 | 0.272 |
| Word type * set size: 5-3 | 7.93 | 13.47 | 0.59 | 0.556 |
|  | | | | |
