# Supplementary material for: A novel multi-word paradigm for investigating semantic context effects in language production
Source: PLoS One. 2020 Apr 10;15(4):e0230439. doi: 10.1371/journal.pone.0230439 (PMC7147796; doi:10.1371/journal.pone.0230439)
Supplement: S4 Appendix — (DOCX) [file pone.0230439.s006.docx]

Appendix D. Combined analysis of reaction times and fixation durations

Table D1. GLMM for the effect of fixation durations on reaction times

| Term | Estimate | SE | t | p | |
| --- | --- | --- | --- | --- | --- |
| Intercept | 865.94 | 9.47 | 91.45 | 0.000 | |
| Picture type: rel-unrel ^a^ | 9.76 | 5.68 | 1.72 | 0.086 | |
| Set size: 4-3 | -11.58 | 5.99 | -1.93 | 0.053 | |
| Set size: 5-3 | -10.20 | 5.86 | -1.74 | 0.082 | |
| Fix_durations (related words) ^b^ | 2.54 | 2.58 | 0.98 | 0.327 | |
| Pic type * set size: 4-3 | -19.33 | 12.70 | -1.52 | 0.128 | |
| Pic type * set size: 5-3 | -1.94 | 13.39 | -0.14 | 0.885 | |
| Pic type * Fix_durations | -5.06 | 6.02 | -0.84 | 0.401 | |
| Set size: 4-3 * Fix_durations | 10.20 | 6.52 | 1.56 | 0.118 | |
| Set size: 5-3 * Fix_durations | 9.54 | 6.42 | 1.49 | 0.137 | |
| Pic type * Set size: 4-3 * Fix_durations | -1.68 | 14.63 | -0.11 | 0.909 | |
| Pic type * Set size: 5-3 * Fix_durations | -8.41 | 12.70 | -0.66 | 0.508 | |
| ^a^ henceforth “pic type”. ^b^ henceforth “fix_durations” | | | | |  |
