# Supplementary material for: A novel multi-word paradigm for investigating semantic context effects in language production
Source: PLoS One. 2020 Apr 10;15(4):e0230439. doi: 10.1371/journal.pone.0230439 (PMC7147796; doi:10.1371/journal.pone.0230439)
Supplement: Attachment — Submitted filename: Response_to_reviewers_rev2.docx [file pone.0230439.s008.docx]

**Reviewer 1**

**Overall impression:**

Overall, I found the revised manuscript still lacking in terms of clear predictions that are theoretically motivated, and conclusions that are well-supported by the data. Below I explain at what points I did not follow the rationale.

Theoretical importance of the novel aspects of the paradigm. The first paragraph of the introduction does a good job in explaining how the novel paradigm is novel in comparison to previous work. However, it is vague in explaining the theoretical importance for the novelty, that is, why is it important to, for example “explore this finding [semantic context effects in naming] further”; or as it put in the general discussion, why it is important “to investigate whether and how semantic interference effects through categorical distractors can be modulated”. What question(s) are left open by previous research that this novel paradigm will now be able to address (e.g., how does the current study address a gap (s) in previous studies)? Below I provide examples where the theoretical rationale and importance for the novel aspects of the paradigm (increasing number of distractors, repetition of pictures, eye-gaze fixation measures) continue to be unclear throughout the manuscript.

**Our response:**

*We thank the reviewer for the feedback and hope that our contributions and answers to the comments below address the reviewer’s concerns. Especially, we have elaborated on the novelty of changing only the number of distractors, but keeping the activation strength stable, to investigate mechanisms of lexical competition. Moreover, we have clarified once more our predictions regarding the eye tracking measures and refer to our exploratory analysis of naming repetition in our paradigm.*

**Comment1:**

1) Increasing numbers of distractors
For example in the introduction (p.19 line 108), the authors provide a vague description of the theoretical importance of “manipulating the extent of lexical activation within a lexical cohort”. Please address if successful (in manipulating the extent of lexical activation within a lexical cohort via increasing numbers of word distractors), what this outcome will support in terms of our understanding of word production dynamics and critically, what the result will refute.

**Our response:**

*According to the Swinging Lexical Network model (Abdel Rahman & Melinger, 2009, 2019), the amount of semantic interference through lexical competition is dependent on (1) the activation strength of lexical competitors and (2) the number of competing items. By keeping the activation strength stable (by using semantically closely related stimuli) in this study we are able to tackle the question whether the number of lexical competitors has a direct influence on the amount of semantic interference. This paragraph is now added to the introduction:*

Introduction, lines 110ff:

**Using a set of closely related entries of a number of lexical cohorts the activation strength per item can be assumed largely homogeneous. Using these sets we parametrically change the number of distractors to investigate, whether this has a direct influence on the amount of semantic interference**.

**Comment 2:**
The ms (p. 19, line 120) mentions that previous work increased distractors in a PWI set up but that this manipulation is [presumably] irrelevant because the words had to be read out loud, not just passively read. Please describe why reading out loud vs. passively is an important manipulation in terms of testing different predictions concerning how word production (semantic interference) proceeds at the semantic and lexical levels.
**Our response:**

*Our main argument for these other studies to differ importantly from our design is the fact that the word stimuli were presented consecutively instead of simultaneously. We agree that reading aloud vs quietly may not be the key difference in modulation between previous and our designs, although differences have been described (Laubrock & Kliegl, 2015). Reading words out loud may activate the full preparatory and motor act of production similar to picture naming, therefore encompassing other effects like repetition priming.*

**Comment 3:**

2) Item repetition
Please provide a more specific rationale to explain the necessity of including repetition in a PWI paradigm (p. 22, line 184): “ we consider more evidence necessary to draw systematic conclusion about the stability of the interference effect in PWI paradigms”. In the introduction as written, it is still unclear how finding an effect of repetition fits or does not fit with theoretical predictions which provide evidence for or against a hypothesis. For example, it could be clearly stated that “if we find that interference increases across repetitions this will support an X interpretation of lexical selection/interference but will not support a Y interpretation. Alternatively, if we find that interference decreases across repetitions this supports a Y interpretation but not X interpretation”. The response exclusion hypothesis is briefly discussed in the general discussion (p. 37, line 514) but two sentences was not enough for me to follow the argument.

**Our response:**

*We have rephrased the quoted paragraph as follows:*

Introduction, lines 183ff:

**In contrast to these paradigms, to our knowledge, for a PWI paradigm changes across repetitions have been formally addressed only in one recent study (Kurtz, Schriefers, Mädebach, & Jescheniak, 2018). Using an auditory PWI design, interference effects are reported to be largely stable across naming repetitions of the same pictures** **with phonological distractors. This stands in contrast to the other paradigms mentioned above, and systematic conclusions about the stability of the interference effect in PWI paradigms can only be tentative at present.**

*Considering the scope of this paper and the lack of previous findings on repetition in PWI, we don’t feel comfortable formulating clear hypotheses about the meaning of a repetition effect for word production models. However, we have elaborated on the discussion of the response-exclusion hypothesis and our opinion of why it is unfitting to our findings:*

Discussion, lines 515ff:

**Notably our findings also contrast with alternative explanations of the origins of semantic interference, specifically the response-exclusion hypothesis (Mahon, Costa, Peterson, Vargas, & Caramazza, 2007). This theory posits that through frequent exposure, task-relevant responses (e.g. names of pictures from the same semantic category) need to be actively excluded from an articulatory output-buffer resulting in delayed naming. In our paradigm, these task-relevant items would include previously named pictures, and previously fixated words that were part of a category word set. However, as we discuss in more detail below, our results show that indeed frequent exposure to the material leads to *faster* naming thus making an explanation of an effortful and therefore inhibitory monitoring mechanism unlikely. .**

**Comment 4:**

The motivation for the repetition factor in this new PWI paradigm is different in the introduction vs. the general discussion. In the general discussion, the authors argue that because repetition affects semantic interference in blocked cyclic and continuous naming, it should be explored in this PWI paradigm variant. However, the repeated exposure in the continuous/blocked cyclic naming paradigm is one of exposure to the same semantic category during naming, not necessarily the same items being named (cf. blocked cyclic paradigm). Thus, I do not clearly follow the theoretical comparison between repeated naming of the same pictures in a PWI paradigm format vs. the repeated retrieval from the same semantic category of different items in continuous/blocked cyclic naming paradigms.

**Our response:**

*In order to avoid a potential misinterpretation of our claims that the repetition effect in PWI and other paradigms might stem from the same origin, we have added a sentence to the discussion for clarification:*

**Note, however, that this sort of cumulative interference results from the repetition of *categories*, not single *items*.** (Discussion, lines 515f.)

*The design of our paradigm and many other PWI paradigms requires the repetition of items due to randomization.* *While researching potential consequences of this design feature, we failed to find reports in the literature. We therefore recommend this to be further addressed by future studies.*

**Comment 5:**

The authors explain that semantic interference disappears after an item is first named because facilitation (during the SR trials) increasingly neutralizes the interference effect (during the SR trials) across repetitions. However, under this interpretation, do the authors predict that facilitation should eventually “win” against interference with multiple repetitions and make the SR condition faster than the UR condition as repetitions increase? The results demonstrate that after the first repetition the SR/UR trials RTs are virtually identical. It is not clear what the predictions were and what the results support.
**Our response:**

*Due to the lack of previous findings on repetition effects in PWI, and the fact that our paradigm is a novel variation of the classical paradigm, we see the inclusion of the factor “repetition” more as an exploratory analysis, and would like to remain with the predictions that are already stated in the Introduction (lines 203ff.)*

**Comment 6:**

3) Eye gaze
Word distractor eye-fixations are introduced in the paradigm to measure “semantic competence”. Semantic competence is referred to as semantic processing abilities or the ability “to establish the semantic relationships between concepts”. However, I still find the rationale confusing. This is because the rationale here is that in neurotypical populations “longer fixation durations…indicate intact semantic processing abilities” but longer fixation durations in clinical populations indicate difficulties to “establish the semantic relationships between concepts”. This logic is the opposite from that used to describe how neurotypical and brain-damaged subjects perform in blocked-cyclic naming where the pattern is assumed to be the same (i.e., longer RTs and/or more errors in the semantically related vs. unrelated blocks for both populations are reflective of the same underlying mechanisms, i.e. semantic interference during naming). Thus, it is unclear how the eye gaze = increased connection strength at the semantic level = better semantic competence argument works.
**Our response:**

*We apologize if our hypotheses regarding the eye tracking measures were still unclear. However, as we describe in the introduction, our assumption about fixation durations to semantically related vs unrelated items reflecting semantic competence is informed by studies on clinical populations (Faria, Race, Kim, & Hillis, 2018; Seckin et al., 2016). These studies showed that impaired semantic knowledge leads to blurry distinctions between semantic categories reflected in longer fixations to foils, i.e. more equal fixation patterns for related and unrelated items. We simply posit that neurotypical young adults have intact semantic knowledge and should therefore should a clear distinct pattern between fixations to related vs. unrelated words. This hypothesis is confirmed by our data.*

**Comment 7:**

Regarding the finding that subjects spent more time looking at words that were semantically related vs. unrelated, the authors conclude that this resolves a debate into the literature as to whether Ss implicitly categorize words when not specifically instructed. However, there is no mention in the manuscript concerning the previous debate concerning this point, how the current results rectify this gap in the literature, nor a clear explanation of how implicit “word categorization” occurs with respect to what is theorized to occur in the PWI paradigm.
**Our response:**
*We may have missed the point and unfortunately fail to find the specific paragraph the reviewer refers to. Probably we should point out that our claims regarding the eye-tracking findings are quite modest and we certainly do not claim to resolve a debate in the literature. Using eye-tracking we simply aimed to assess a measure of how participants process the set of words presented. From our review of literature on participants with impaired language and semantic abilities we concluded that the implicit recognition of a cohort can be deduced from a longer fixation on related compared to unrelated words. Our question was on how the intensity of processing is also augmented by longer fixation (likely indicating a more intense processing). Here our finding is that implicit categorization seems to have no further effect on semantic interference (i.e. there is no correlation between fixation durations on semantically related words and reaction times).*

**Comment 8:**

Minor point:
Because 1) the design included a repetition component in order to increase activation, 2) the authors report and interpret the interaction between semantic relatedness (SR/UR) and naming repetitions (times points 2-6), and 3) the general discussion refers to the change (or lack thereof) in the SR/UR conditions across repetitions, please include (supplemental or otherwise) the figure including both SR and UR conditions across all repetitions.

**Our response:**

*The figure below is now included in the appendix of the paper.*

**Literature**

Abdel Rahman, R., & Melinger, A. (2009). Semantic context effects in language production: A swinging lexical network proposal and a review. *Language and Cognitive Processes*, *24*(5), 713–734. https://doi.org/10.1080/01690960802597250

Abdel Rahman, R., & Melinger, A. (2019). Semantic processing during language production: an update of the swinging lexical network. *Language, Cognition and Neuroscience*, *34*(9), 1176–1192. https://doi.org/10.1080/23273798.2019.1599970

Faria, A. V., Race, D., Kim, K., & Hillis, A. E. (2018). The eyes reveal uncertainty about object distinctions in semantic variant primary progressive aphasia. *Cortex*, *103*, 372–381. https://doi.org/10.1016/j.cortex.2018.03.023

Kurtz, F., Schriefers, H., Mädebach, A., & Jescheniak, J. D. (2018). Incremental learning in word production: Tracing the fate of non-selected alternative picture names. *Journal of Experimental Psychology: Human Perception and Performance*, *44*(10), 1586–1602. https://doi.org/10.1037/xhp0000558

Laubrock, J., & Kliegl, R. (2015). The eye-voice span during reading aloud. *Frontiers in Psychology*, *6*, 1432. https://doi.org/10.3389/fpsyg.2015.01432

Mahon, B. Z., Costa, A., Peterson, R., Vargas, K. A., & Caramazza, A. (2007). Lexical Selection Is Not by Competition: A Reinterpretation of Semantic Interference and Facilitation Effects in the Picture-Word Interference Paradigm. *Journal of Experimental Psychology: Learning Memory and Cognition*, *33*(3), 503–535. https://doi.org/10.1037/0278-7393.33.3.503

Seckin, M., Mesulam, M. M., Voss, J. L., Huang, W., Rogalski, E. J., & Hurley, R. S. (2016). Am I looking at a cat or a dog? Gaze in the semantic variant of primary progressive aphasia is subject to excessive taxonomic capture. *Journal of Neurolinguistics*, *37*, 68–81. https://doi.org/10.1016/j.jneuroling.2015.09.003
